# Dendritic cells play no significant role in the laser-induced choroidal neovascularization model

**DOI:** 10.1038/s41598-021-96704-x

**Published:** 2021-08-26

**Authors:** Steven Droho, Harris Perlman, Jeremy A. Lavine

**Affiliations:** 1 Department of Ophthalmology, Feinberg School of Medicine, Northwestern University, Chicago, IL USA; 2 Division of Rheumatology, Department of Medicine, Feinberg School of Medicine, Northwestern University, Chicago, IL USA

**Keywords:** Dendritic cells, Macular degeneration

## Abstract

Age-related macular degeneration (AMD) is genetically associated with complement. Dendritic cells (DCs) play key roles during innate and adaptive immunity, and express complement components and their receptors. We investigated ocular DC heterogeneity and the role of DCs in the laser-induced choroidal neovascularization (CNV) model. In order to determine the function of DCs, we used two models of DC deficiency: the *Flt3*^*−/−*^ and *Flt3l*^*−/−*^ mouse. We identified three types of ocular DCs: plasmacytoid DC, classical DC-1, and classical DC-2. At steady-state, classical DCs were found in the iris and choroid but were not detectable in the retina. Plasmacytoid DCs existed at very low levels in iris, choroid, and retina. After laser injury, the number of each DC subset was up-regulated in the choroid and retina. In *Flt3*^*−/−*^ mice, we found reduced numbers of classical DCs at steady-state, but each DC subset equally increased after laser injury between wildtype and *Flt3*^*−/−*^ mice. In *Flt3l*^*−/−*^ mice, each DC subsets was severely reduced after laser injury. Neither *Flt3*^*−/−*^ or *Flt3l*^*−/−*^ mice demonstrated reduced CNV area compared to wildtype mice. DCs do not play any significant role during the laser-induced CNV model of neovascular AMD.

## Introduction

Age-related macular degeneration (AMD) is one of the most common causes of vision loss. Two forms of AMD exist: non-neovascular and neovascular AMD (nAMD). Drusen, which are inflammatory lipoprotein deposits, are the pathognomonic feature of non-neovascular AMD. Neovascular AMD is characterized by a process termed choroidal neovascularization (CNV) where aberrant and destructive angiogenesis from the choroidal vasculature, theoretically trigged by drusen, invades into the sub-retinal pigment epithelium (RPE) or sub-retinal space. Drusen are composed of multiple features, including complement factors and components^1^, which are genetically associated with AMD^2–4^. Dendritic cells (DCs) express complement components and receptors for activated complement proteins^5^, suggesting that DCs may play a role in AMD pathogenesis.

Dendritic cells (DCs) demonstrate essential and diverse functions during innate and adaptive immunity. Currently, DCs can be divided into plasmacytoid DCs and classical (or conventional) DCs (cDC). Plasmacytoid DCs express type 1 interferons during viral infection^6^, and express the universal DC markers CD11c and MHCII in addition to unique DC markers which are normally expressed on lymphocytes including CD4, CD8, and B220^7^. Classical DCs can be further sub-divided into cDC-1 and cDC-2 subsets. Classical DC-1 cells present antigens primarily to CD8^+^ T-cells to combat intracellular pathogens and cancer; cDC-2 display antigens to CD4^+^ T-cells to control the immune response to extracellular pathogens, allergens, and parasites^6^. Classical DC-1 and DC-2 subsets express MHCII and CD11c, do not express the macrophage/monocyte markers Ly6C or CD64, and can be discriminated by CD11b^−^Xcr1^+^ cDC-1 versus CD11b^+^Xcr1^−^ cDC-2 markers^8^.

Prior studies show that intravenous injection of MHCII^+^CD11c^+^ bone marrow-derived cells increased CNV area during the laser-induced CNV model^9^. However, macrophages promote CNV and are capable of expressing MHCII and CD11c^10^. Thus, the role of DCs and their subtypes remains unclear in AMD pathogenesis. In this study, we characterized DC heterogeneity in the mouse eye, and tested two models of DC deficiency in the laser-induced CNV model. We found that plasmacytoid DCs, cDC-1, and cDC-2 subtypes were detected in the mouse choroid, and all 3 subsets were increased during the laser-induced CNV model. Additionally, DC deficiency did not result in a reduction in CNV area. Therefore, we found that DCs do not play a significant role during the laser-induced CNV mouse model of nAMD.

## Results

### Multi-parameter flow cytometry of ocular DCs

**Figure 1:**
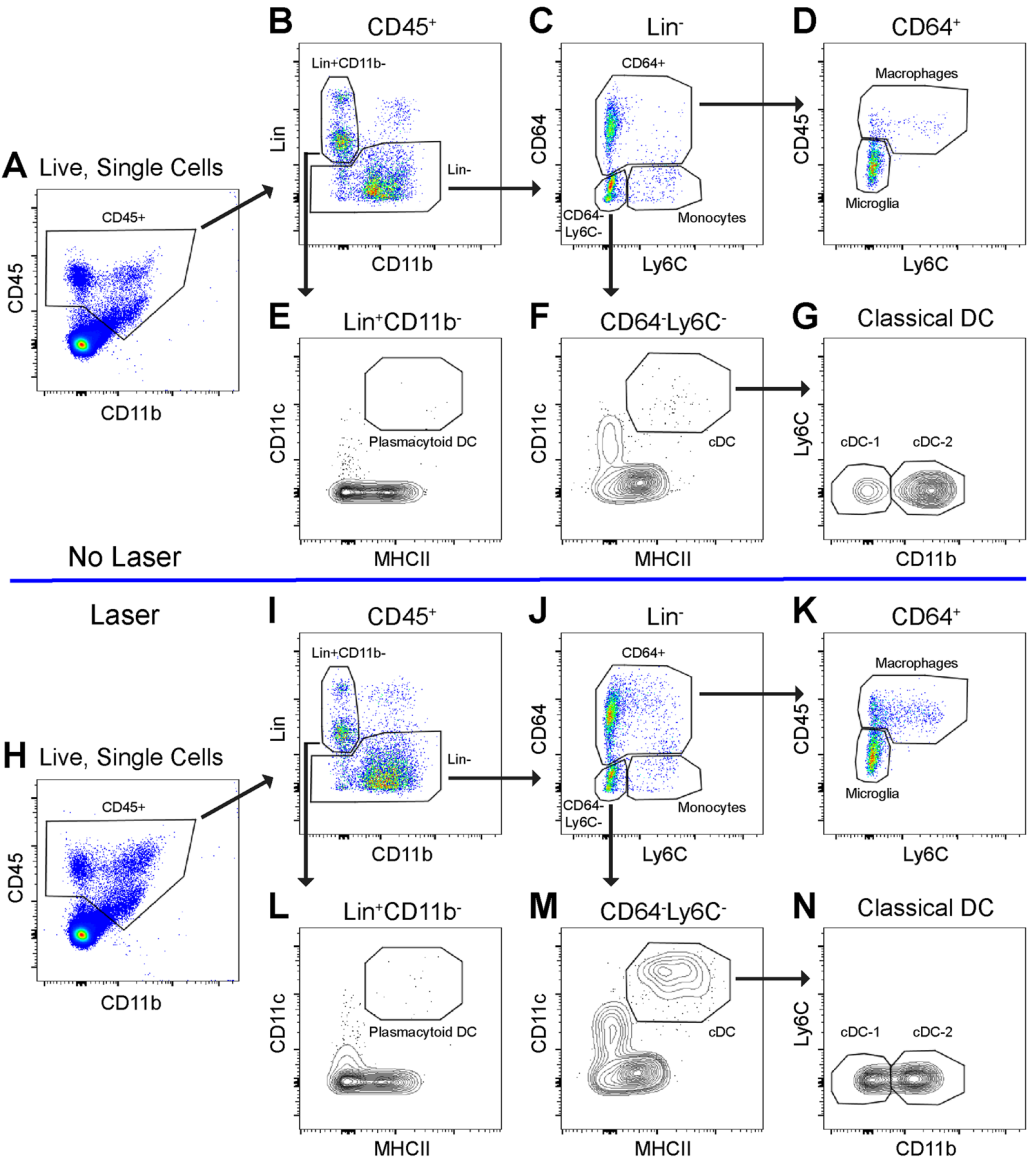
Gating strategy to identify DC heterogeneity in control (No Laser, **A**–**G**) and laser-treated mice (**H**–**N**). (**A**, **H**) CD45^+^ cells were gated forward. (**B**, **I**) Pseudocolor plot of Lineage (Lin: CD4, CD8, B220, SiglecF, Ly6G, NK1.1) versus CD11b delineated Lin^−^ mononuclear phagocytes and Lin^+^CD11b^−^ lymphocytes and plasmacytoid DCs. (**C**, **J**) Pseudocolor plot of Lin^−^ cells discriminated CD64^+^ cells, CD64^−^Ly6C^+^ monocytes, and CD64^−^Ly6C^−^ cells. D,K: CD64^+^ cells from C,J were gated forward, CD45^dim^ microglia and CD45^high^Ly6C^mixed^ macrophages were defined. (**E**, **L**) MHCII versus CD11c contour plot of Lin^+^CD11b^−^ cells identified MHCII^+^CD11c^+^ plasmacytoid DCs. (**F, M**) MHCII versus CD11c contour plot of CD64^−^Ly6C^−^ cells delineated MHCII^+^CD11c^+^ classical DCs (cDC). (**G, M**) Classical DC-1 and DC-2 cells were defined as CD11b^−^ and CD11b^+^, respectively.

We used multi-parameter flow cytometry to analyze dendritic cell heterogeneity in murine eyes at steady state and after laser-induced CNV. Whole mouse eyes were dissected to remove conjunctiva, orbital tissue, extraocular muscles, and optic nerve. Cornea, sclera, iris, ciliary body, lens, vitreous, retina, and choroid were chemically and mechanically digested for multi-parameter flow cytometry at steady-state or 3 days post laser injury. CD45^+^ cells were identified from singlet, live cells and gated forward (Fig. 1A, H). T-cells (CD4 and CD8), B-cells (B220), NK cells (NK1.1), eosinophils (SiglecF), and neutrophils (Ly6G) were excluded using a lineage (Lin) gate (Fig. 1B, I). Lin^+^CD11b^−^ cells, which include lymphocytes and plasmacytoid DCs, which can express CD4, CD8, and/or B220^7^, were gated forward (Fig. 1B, I). Plasmacytoid DCs were delineated from Lin^+^CD11b^−^ cells as MHCII^+^CD11c^+^ (Fig. 1E, L). From CD45^+^Lin^−^ cells, CD64^+^ cells were gated forward (Fig. 1C, J); microglia were CD45^dim^Ly6C^−^ and macrophages were CD45^high^Ly6C^mixed^ (Fig. 1D, K). From CD45^+^Lin^−^ cells, the CD64^−^Ly6C^−^ population was gated forward (Fig. 1C, J), and MHCII^+^CD11c^+^ cells were defined as classical DC (cDC, Fig. 1F, M). Both CD11b^−^ cDC-1 and CD11b^+^ cDC-2 subsets were found (Fig. 1G, N). These data show that we can detect 3 DC population in the mouse eye. Please see our recent publication for our fluorescence minus one controls^11^.

**Figure 2:**
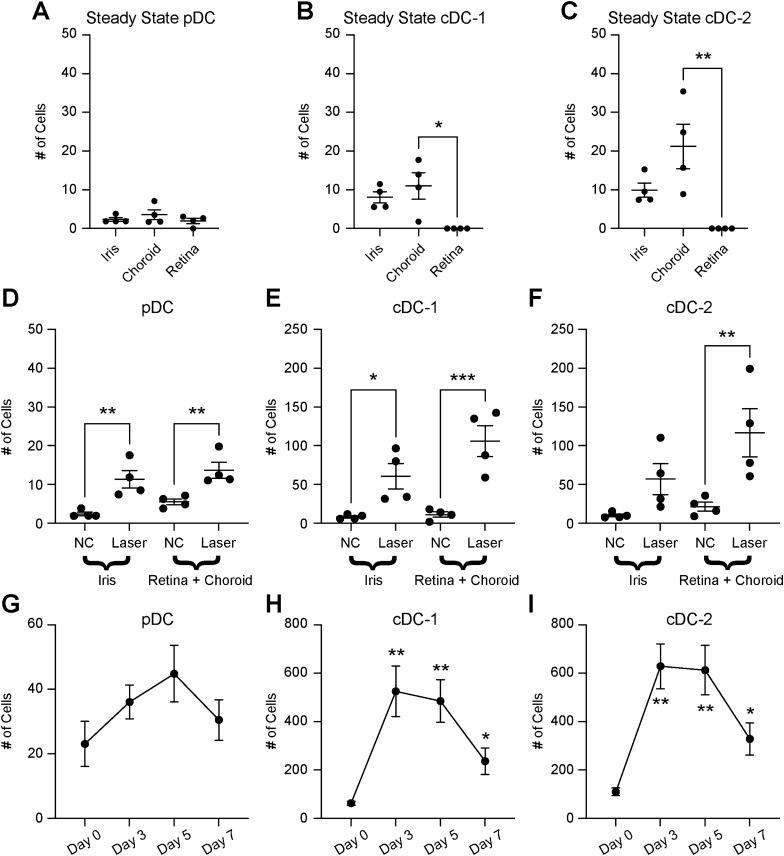
Each DC subtype was increased in the retina and choroid after laser injury. Number of plasmacytoid DCs (pDC, **A**), classical DC-1 (cDC-1), and classical DC-2 (cDC-2) identified in each ocular tissue at steady state. Number of pDC (**D**), cDC-1 (**E**), and cDC-2 (**F**) in either iris or retina and choroid in untreated (NC) or laser-treated mice. Number of pDC (**G**), c-DC1 (**H**), and c-DC2 (**I**) over time after laser injury (N = 9 per group). *p < 0.05; **p < 0.01; ***p < 0.001.

To identify the ocular sub-compartment of each DC subset, we dissected eyes into iris, choroid, and retina tissues. Each ocular tissue underwent multi-parameter flow cytometry with the gating strategy described in Fig. 1. At steady-state, we found similarly low numbers of plasmacytoid DCs in each tissue (Fig. 2A). We found both cDC-1 and cDC-2 populations in iris and choroid, but could not detect cDCs in the retina (Fig. 2B,C). Since the laser-induced inflammatory lesion exists in the subretinal space, we analyzed DC heterogeneity in both retina and choroid tissues together on Day 3 after laser injury. Plasmacytoid DCs increased in both iris (4.8-fold, *p* < 0.01) and choroid/retina (2.5-fold, *p* < 0.01) after laser injury (Fig. 2D). The number of cDC-1 cells was up-regulated by 7.5-fold (*p* < 0.05) in iris and 9.6-fold in choroid/retina (*p* < 0.001) after laser injury (Fig. 2E). The cDC-2 population enlarged 5.5-fold (*p* < 0.01) in choroid/retina, but only a trend toward increased cDC-2 cells was found in the iris after laser treatment (Fig. 2F). These data demonstrate that each DC subset expands in the posterior segment after laser injury, while a similar but smaller increase is observed in the iris.

We previously published that macrophage infiltration peaks on Day 3 after laser injury^10^. We performed multi-parameter flow cytometry (identical to Fig. 1) on Day 3, Day 5, and Day 7 to investigate tissue infiltration of each DC subtype. This study was performed on whole eye samples (cornea, sclera, iris, ciliary body, lens, vitreous, retina, and choroid) to increase DC capture, maximize rigor and reproducibility, and minimize error created from uneven tissue dissections. We found that plasmacytoid DCs trended upward with a peak between Day 3–5 (Fig. 2G). Classical DC-1 cells increased 8.4-fold on Day 3 (*p* < 0.01 vs Day 0), 7.7-fold on Day 5 (*p* < 0.01 vs Day 0), and 3.8-fold on Day 7 (*p* < 0.05 vs Day 0, Fig. 2H). The cDC-2 population enlarged 5.7-fold on Day 3 (*p* < 0.01 vs Day 0), 5.6-fold on Day 5 (*p* < 0.01 vs Day 0), and 3.0-fold on Day 7 (*p* < 0.05 vs Day 0, Fig. 2I). These data demonstrate that the peak of DC infiltration is Day 3 similar to macrophages in the laser-induced CNV model.

### Effect of Flt3 deficiency on CNV and DCs

**Figure 3:**
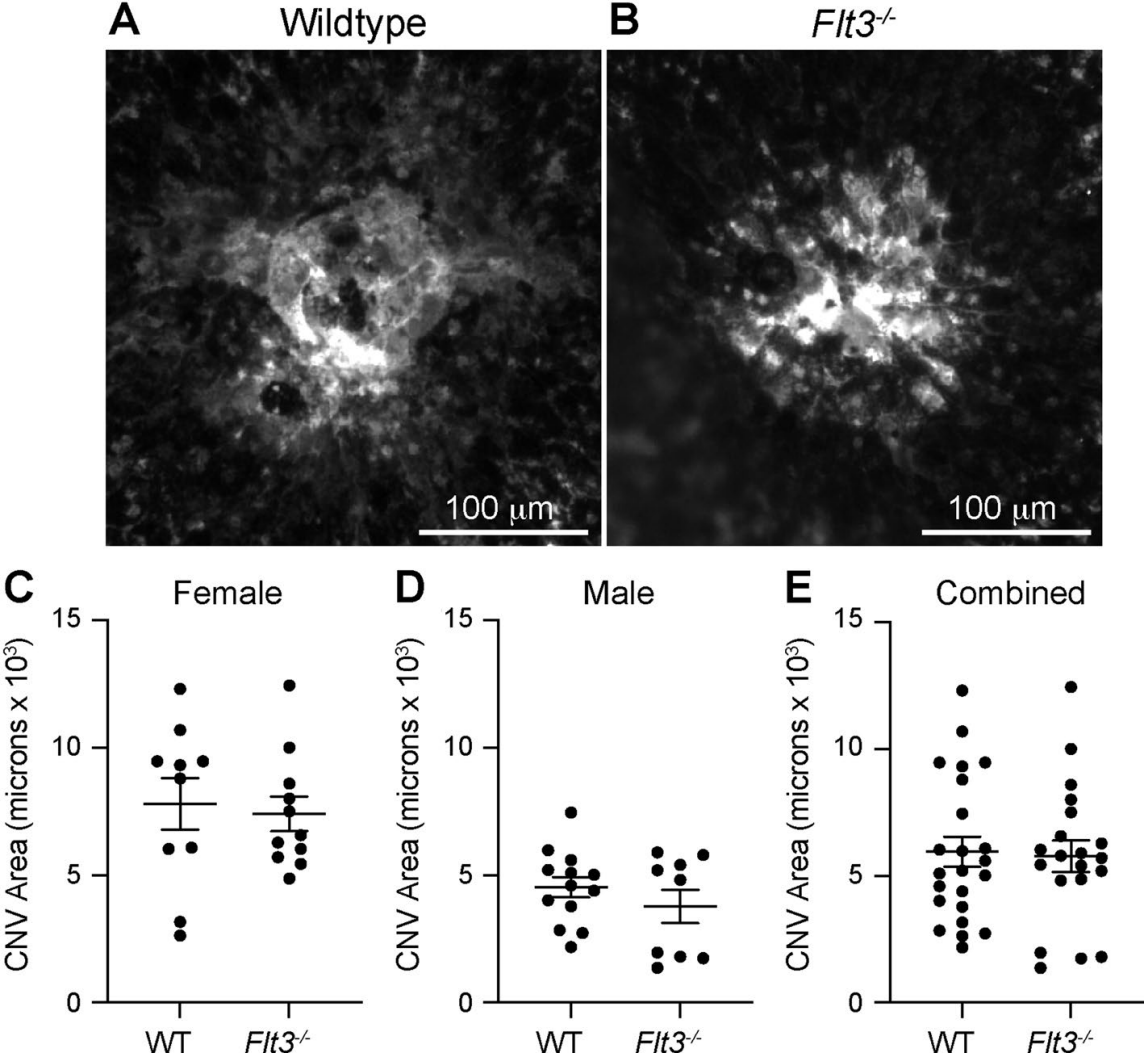
*Flt3*^*−/−*^ mice showed no change in laser-induced CNV area. (**A, B**) representative images of ICAM-2 stained laser-induced CNV lesions. CNV area was equal between wildtype (WT) and *Flt3*^*−/−*^ mice female (**C**, N = 10–11 per group), male (**D**, N = 9–13 per group), and all mice (**E**, N = 20–23 per group).

In order to investigate the function of DCs during laser-induced CNV, we first investigated the *Flt3*^*−/−*^ mouse. Fms-like tyrosine kinase 3 (Flt3) is a cell surface receptor that is necessary to maintain tissue resident DC populations via homeostatic DC proliferation^12^. Wildtype and *Flt3*^*−/−*^ male and female mice were treated with laser to break Bruch’s membrane and induce CNV formation. On Day 14 after laser injury, we stained choroidal wholemounts with ICAM-2 to measure CNV area. We found no change in CNV area as a result of *Flt3* deficiency in female (7798 mm^2^ [wildtype] vs 7406 µm^2^ [*Flt3*^*−/−*^], *p* = 0.75, Fig. 3C), male (4531 µm^2^ vs 3777 µm^2^, *p* = 0.31, Fig. 3D), or when all mice were combined (5951 µm^2^ vs 5773 µm^2^, *p* = 0.84, Fig. 3E).

**Figure 4:**
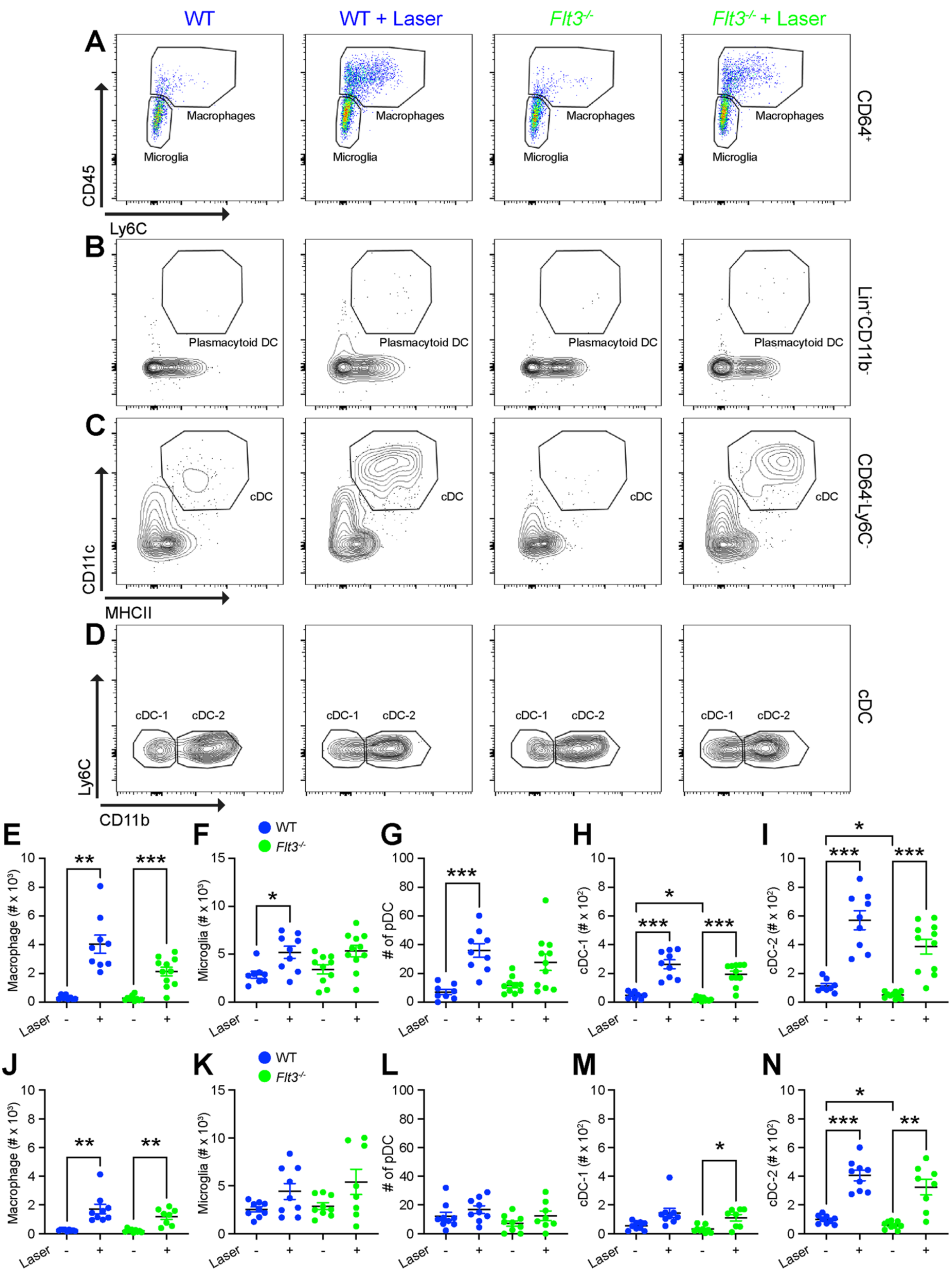
*Flt3*^*−/−*^ mice demonstrated reduced DC numbers at steady-state. (**A**) Representative CD45 versus Ly6C pseudocolor plots of CD64^+^ cells from control and laser-treated wildtype (WT) and *Flt3*^*−/−*^ mice to identify microglia and macrophages. (**B**, **C**) Representative CD11c versus MHCII contour plots of Lin^−^CD11b^+^ cells (**B**) and CD64^−^Ly6C^−^ cells (**C**) to define plasmacytoid DC (**B**) and classical DC (cDC, **C**). D: Representative Ly6C versus CD11b contour plot of cDCs to discriminate CD11b^−^ cDC-1 and CD11b^+^ cDC-2 from control and laser-treated wildtype (WT) and *Flt3*^*−/−*^ mice. (**E**–**I**) Number of macrophages, microglia, and DC subtypes from wildtype (WT, blue) and *Flt3*^*−/−*^ mice (green) female mice (N = 8–10 per group). J-N: Number of macrophages, microglia, and DC subtypes from wildtype (WT, blue) and *Flt3*^*−/−*^ mice (green) male mice (N = 8–9 per group). (**E**, **J**) Macrophage numbers were increased after laser in both genotypes and sexes. (**F, K**) Microglia numbers were relatively unchanged by genotype or laser in both sexes. (**G, L**) Plasmacytoid DCs were up-regulated by laser in female WT mice only. (**H, M**) Steady-state cDC-1 cells were decreased in female *Flt3*^*−/−*^ mice. Classical DC-1 numbers elevated after laser in both genotypes and sexes. I-N: Steady-state cDC-2 were reduced in male and female *Flt3*^*−/−*^ mice. Laser increased cDC-2 cells in both genotypes and sexes. *p < 0.05, **p < 0.01, ***p < 0.001.

We next used multi-parameter flow cytometry to measure DC heterogeneity in the context of *Flt3* deficiency. Since DC numbers per mouse were low and we found no significant difference in the response of each ocular sub-compartment to laser injury (Fig. 2), we performed our DC heterogeneity analysis in whole mouse eyes (cornea, sclera, iris, ciliary body, lens, vitreous, retina, and choroid). We chose Day 3 for multi-parameter flow cytometry studies, as it is the peak of both macrophage^10^ and DC infiltration (Fig. 2G–I) after laser injury. In females, macrophage numbers increased 12.8-fold (*p* < 0.01) in wildtype and 8.0-fold (*p* < 0.001) in *Flt3*^*−/−*^ mice after laser injury, with no significant difference between groups (Fig. 4A, E). Similarly, in males, macrophages expanded 5.9–7.3 fold after laser injury (*p* < 0.01 for both), with no significant differences between groups (Fig. 4J). Microglia numbers remained relatively unchanged regardless of sex, laser, or genotype (Fig. 4A, F, K). In female mice, plasmacytoid DCs increased 5.1-fold (*p* < 0.001) in wildtype mice, but were not significantly increased in *Flt3*^*−/−*^ mice (Fig. 4B, G). Plasmacytoid DC numbers were unchanged by genotype or laser in male mice (Fig. 4L). At steady-state, cDC-1 numbers decreased by 58% in female *Flt3*^*−/−*^ mice (*p* < 0.05, Fig. 4C, D, H), and were unchanged in male mice (Fig. 4M). After laser injury, the c-DC1 population was up-regulated by 5.6–9.7 fold (*p* < 0.001 for both, Fig. 4C, D, H) in female mice, and 2.6–3.5 fold (*p* < 0.05 for *Flt3*^*−/−*^) in male mice (Fig. 4M) with no difference between genotypes . The steady-state cDC-2 population was significantly reduced in both sexes by 40–56% in *Flt3*^*−/−*^ mice (*p* < 0.05 for both, Fig. 4C, D, I, N). After laser injury, cDC-2 cells expanded by 5.1–7.8 fold in female mice (*p* < 0.001 for both, Fig. 4C, D, I) and 4.1–5.5 fold in male mice (*p* < 0.01 for both, Fig. 4N) with no significant differences between wildtype and *Flt3*^*−/−*^ mice. These data display that *Flt3*^*−/−*^ mice have reduced DCs at steady-state but are still capable of increasing their DC populations after laser injury.

### Effect of Flt3 ligand deficiency on CNV and DCs

**Figure 5:**
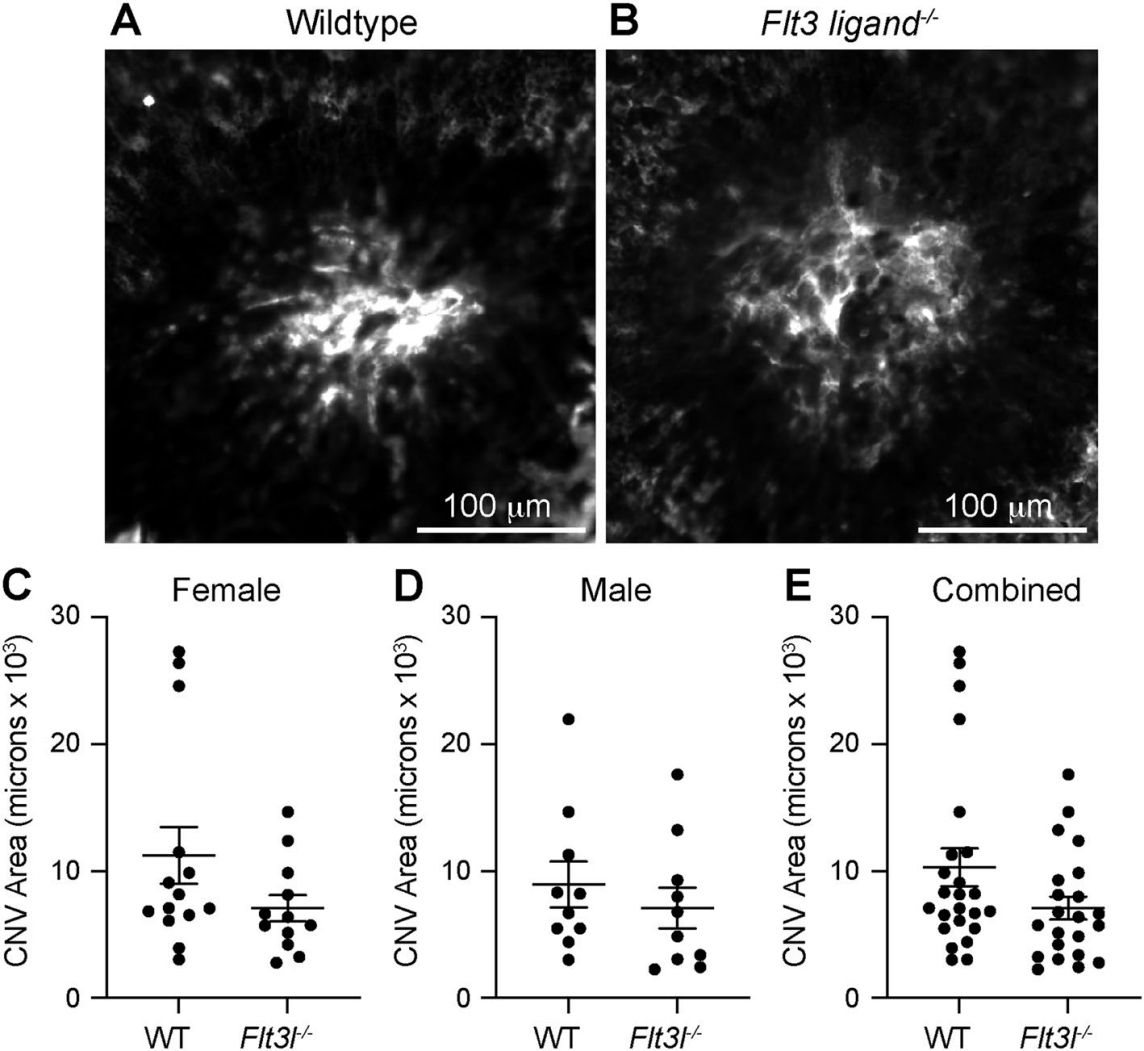
*Flt3l*^*−/−*^ mice showed no change in laser-induced CNV area. (**A, B**) representative images of ICAM-2 stained laser-induced CNV lesions. CNV area was equal between wildtype (WT) and *Flt3l*^*−/−*^ mice female (**C**, N = 12–14 per group), male (**D**, N = 10 per group), and all mice (**E**, N = 22–24 per group).

We next investigated the *Flt3 ligand* deficient (*Flt3l*^*−/−*^) mouse, a more severe model of dendritic cell deficiency^13^. Wildtype and *Flt3l*^*−/−*^ male and female mice underwent laser-induced CNV. On Day 14 after laser, we stained choroidal wholemounts to measure CNV area. We found a trend toward reduced CNV area in female *Flt3l*^*−/−*^ mice (11,249 µm^2^ [wildtype] vs 7077 µm^2^ [*Flt3l*^*−/−*^], *p* = 0.12, Fig. 5C), no change in male mice (8955 µm^2^ vs 7092 µm^2^, *p* = 0.45, Fig. 5D), and no significant difference when all mice were combined (10,293 µm^2^ vs 7084 µm^2^, *p* = 0.08, Fig. 5E).

**Figure 6:**
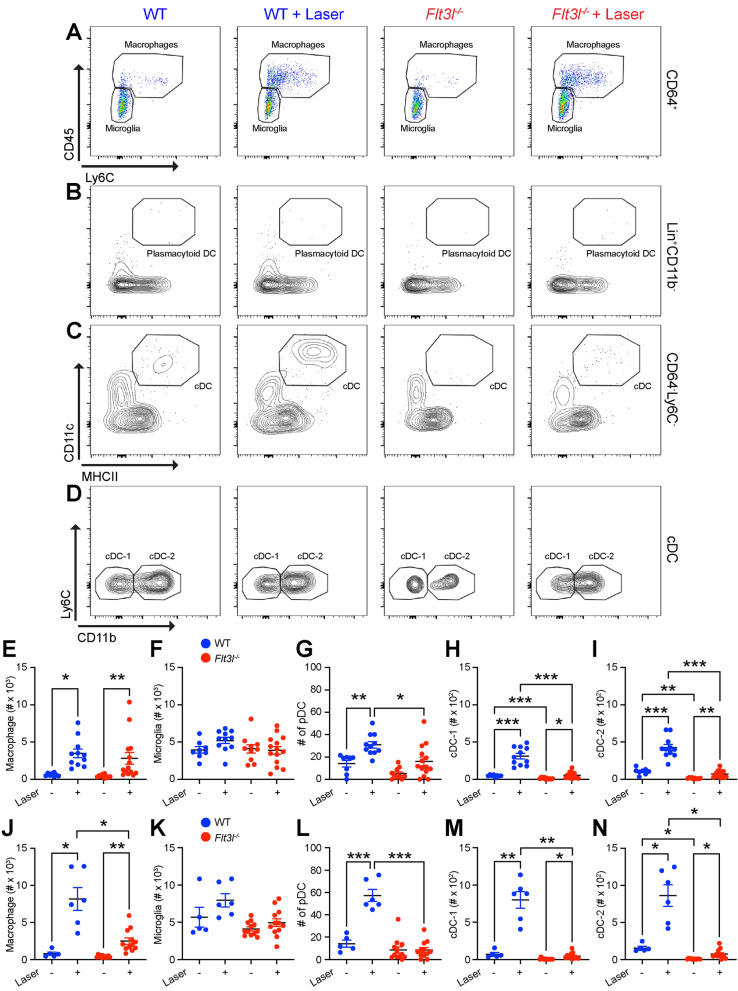
DC numbers in *Flt3l*^*−/−*^ mice were ablated at steady-state and after laser injury. (**A**) Representative CD45 versus Ly6C pseudocolor plots of CD64^+^ cells from control and laser-treated wildtype (WT) and *Flt3l*^*−/−*^ mice to identify microglia and macrophages. (**B**, **C**) Representative CD11c versus MHCII contour plots of Lin^−^CD11b^+^ cells (**B**) and CD64^−^Ly6C^−^ cells (**C**) to define plasmacytoid DC (**B**) and classical DC (cDC, **C**). (**D**) Representative Ly6C versus CD11b contour plot of cDCs to discriminate CD11b^−^ cDC-1 and CD11b^+^ cDC-2 from control and laser-treated wildtype (WT) and *Flt3l*^*−/−*^ mice. E-I: Number of macrophages, microglia, and DC subtypes from wildtype (WT, blue) and *Flt3l*^*−/−*^ mice (red) female mice (N = 8–14 per group). (**J**–**N**) Number of macrophages, microglia, and DC subtypes from wildtype (WT, blue) and *Flt3*^*−/−*^ mice (red) male mice (N = 6–12 per group). (**E**, **J**) Macrophage numbers were increased after laser in both genotypes and sexes. (**F, K**) Microglia numbers were relatively unchanged by genotype or laser in both sexes. (**G**, **L**) Plasmacytoid DCs were up-regulated by laser in WT mice of both sexes but remained unchanged in *Flt3l*^*−/−*^ mice after laser injury. (**H**, **M**) cDC-1 cells were increased by laser in WT mice but severely reduced at both steady-state and after laser treatment in male and female *Flt3l*^*−/−*^ mice. (**I**–**N**) cDC-2 cells were up-regulated by laser in WT mice but ablated at both steady-state and after laser treatment in male and female *Flt3l*^*−/−*^ mice. *p < 0.05, **p < 0.01, ***p < 0.001.

In order to characterize the effect of *Flt3l* deficiency on DC heterogeneity, we performed multi-parameter flow cytometry on Day 3 after laser injury. In female mice, macrophages numbers increased 5.6-fold in wildtype (*p* < 0.05) and 6.6-fold in *Flt3l*^*−/−*^ mice after laser treatment (*p* < 0.01) with no difference between groups (Fig. 6A, E). In male mice, macrophages expanded by 10.2-fold in wildtype mice (*p* < 0.05) and 5.8-fold in *Flt3l*^*−/−*^ mice (*p* < 0.01) in response to laser, which was significantly reduced compared to wildtype laser-treated mice (*p* < 0.05, Fig. 6J). Microglia numbers were unchanged by laser, genotype, or sex (Fig. 6A, F, K). In female and male wildtype mice, plasmacytoid DC numbers elevated after laser treatment by 2.2-fold (*p* < 0.01, Fig. 4B, G) and 4.0-fold (*p* < 0.001, Fig. 4L) respectively. In female and mice *Flt3l*^*−/−*^ mice, plasmacytoid DCs were unchanged by laser and significantly decreased compared to wildtype-lasered mice (Fig. 6B, G, L). At steady-state, cDC-1 and c-DC2 cells were reduced by 76–87% in female (*p* < 0.01 for both, Fig. 6C, D, H, I) and 89–95% in male mice (*p* < 0.05 for c-DC2, Fig. 6M, N). Both cDC-1 and cDC-2 populations were significantly increased by laser in female and male wildtype mice (Fig. 6C, D, H, I, M, N). In *Flt3l*^*−/−*^ mice, however, cDC-1 and cDC-2 cells were nearly completely ablated with 83% reductions in female (*p* < 0.001, Fig. 6C, D, 6H, I) and 91–94% declines in male mice (*p* < 0.05, Fig. 6M, N). These data indicate that *Flt3l*^*−/−*^ mice have severe deficiency of all DC subsets and no significant difference in CNV area, indicating that DCs play no significant role during the laser-induced CNV model.

## Discussion

In this study, we show that DC heterogeneity exists in the eye, where we were able to detect classical DCs in the uvea, including the choroid and iris, but not in the retina (Fig. 2). Additionally, we found low numbers of plasmacytoid DCs in iris, choroid, and retina (Fig. 2). After laser injury, the number of each DC subtype increased in the posterior segment (Fig. 2). In order to determine the role of DCs during laser-induced CNV, we tested two models of DC deficiency. *Flt3*^*−/−*^ mice displayed a classical DC deficit at steady-state (Fig. 4), which resulted in no change in laser-induced CNV area (Fig. 3). *Flt3l*^*−/−*^ mice demonstrated a severe DC absence at both steady-state and after laser injury (Fig. 6), which caused no significant reduction in laser-induced CNV area (Fig. 5). Therefore, DCs do not play a significant role in the laser-induced CNV model of nAMD.

Our results contradict a prior report where DCs were able to augment the laser-induced CNV model. In this study from 2008, bone marrow-derived cells were cultured for 9 days in the presence of granulocyte–macrophage colony-stimulating factor (Csf2) to mature MHCII^+^CD11c^+^ cells^9^. These MHCII^+^CD11c^+^ cells were injected systemically, and larger laser-induced CNV lesions were observed. Since this publication in 2008, several markers, including CD64, were found to reliably distinguish macrophages and DCs^14^. We have previously published, using CD64 to discriminate macrophages from DCs, that MHCII^+^CD11c^+^ macrophage numbers are up-regulated during laser-induced CNV and express a pro-angiogenic transcriptome^10^. Therefore, the MHCII^+^CD11c^+^ population investigated by Nakai et al.^9^ likely included both macrophages and DCs, explaining the discrepancy in our results.

*Flt3*^*−/−*^ mice and intravitreal pharmacologic Flt3 blockade were previously shown to inhibit laser-induced CNV by ~ 40%, which we were not able to replicate^15^. The pharmacologic Flt3 inhibitor AC220 is specific at nanomolar levels, but the authors used 0.1–10 mg/ml (0.18–18 mM). At 6 orders of magnitude above specificity, AC220 has many off target effects including inhibition of platelet-derived growth factor receptors and colony stimulating factor 1 (Csf1) receptor^16^, which both can stimulate choroidal angiogenesis^17–19^. In addition, experiments in this prior publication were performed at 4–8 weeks of age, but the sex and genetic background of the *Flt3*^*−/−*^ mice were not reported. Our experiments were performed on both male (N = 9–13 per groups) and female (N = 10–11 per groups) mice, at 10–12 weeks of age, and the *Flt3*^*−/−*^ mice used in our study were on a C57BL/6 J background (Fig. 3). These discrepancies could explain the differences in results.

We were initially surprised that *Flt3*^*−/−*^ mice demonstrated DC deficits at steady-state only (Fig. 4), while *Flt3l*^*−/−*^ mice showed severe DC deficiencies at both steady-state and in response to laser injury (Fig. 6). However, *Flt3*^*−/−*^ mice can exhibit an enhanced response to Csf1, leading to DC development^20^. Since Csf1 expression increases after laser injury^19^, Csf1-dependent signaling is a possible compensatory mechanism for DC maturation in *Flt3*^*−/−*^ mice, explaining why *Flt3*^*−/−*^ mice have increased DCs after laser injury.

A major limitation of our study is that it is very difficult to prove a negative hypothesis. We tested a single model of nAMD: the laser-induced CNV model. Additionally, we tested two models of DC deficiency: the *Flt3*^*−/−*^ and *Flt3l*^*−/−*^ mice. It is possible that DCs play a role in other models of nAMD like the *Vldlr*^*−/−*^ mouse. It is also possible that another model of DC deficiency could display a phenotype in the laser-induced CNV model. However, given that multiple other cell types including endothelial cells and macrophages express complement proteins and receptors^21^, it is more likely that the association between complement and AMD is not through DCs.

In summary, we were able to detect plasmacytoid DCs, classical DC-1, and classical DC-2 subsets in the mouse choroid, and each subset was increased after laser injury. In the *Flt3*^*−/−*^ mouse model of steady-state DC deficiency, no change in laser-induced CNV area was detected. Finally, in the *Flt3l*^*−/−*^ mouse model of severe DC deficiency at both steady-state and after laser injury, no significant difference in laser-induced CNV was found. Therefore, DCs play no significant role in the laser-induced CNV model.

## Methods

### Animals

Breeding pairs of wildtype (C57BL6/J; #000664) mice were obtained from Jackson Labs (Bar Harbor, ME). *Flt3*^*−/−*^ mice^22^ were a gift from Kenneth M. Murphy (Washington University, St. Louis, MO) who received them from E. Camilla Forsberg (University of California, Santa Cruz, Santa Cruz, CA). *Flt3l*^*−/−*^ mice were a gift from Kenneth M. Murphy who received them from Jackson Labs (C57BL/6-Flt3l^tm1Imx^/TacMmjax). Mice were bred in-house and maintained in a pathogen-free barrier facility within Northwestern University’s Center for Comparative Medicine. One complete litter from each breeding pair was genotyped to confirm the correct genotype and the absence of the RD8 allele (*Crb1-*). Genotyping services were performed by Transnetyx (Cordova, TN). All experiments were conducted in accordance with the ARRIVE guidelines, were approved by the Northwestern University Institutional Animal Care and Use Committee, and were performed in accordance with the relevant guidelines and regulations.

### Laser-induced CNV

Male and female 10–12 week old mice underwent laser treatment as previously described^23^. Briefly, mice were anesthetized with ketamine/xylazine (Akorn, Lake Forest, IL) and received 1 mg/kg subcutaneous injection of Meloxicam (Henry Schein Animal Health, Melville, NY). Eyes were anesthetized, dilated, and a cover slip was coupled to the cornea with Gonak (Akorn) for slit lamp biomicroscopy and laser. Four (immunofluorescence) or eight (flow cytometry, to increase inflammatory cell numbers) focal burns (75 μm, 100–120 mW, 100 ms) were administered in each eye using a 532 nm argon ophthalmic laser (IRIDEX, Mountain View, CA) via a slit lamp (Zeiss, Oberkochen, Germany).

### Immunofluorescence

Eyes were treated as previously described^23^. Briefly, mice were sacrificed 14 days after laser injury. Eyes were enucleated, fixed for 1 h in 1% paraformaldehyde (#15713-S, Electron Microscopy Sciences, Hatfield, PA) at room temperature, washed in PBS, and dissected to remove conjunctiva, cornea, iris, ciliary body, lens, and retina leaving a posterior eye cup of RPE, choroid, and sclera. Eye cups were blocked in Tris buffered saline (TBS) + 5% Donkey serum (S30, Sigma-Aldrich), incubated with an anti-ICAM-2 primary antibody (1:500, Table 1), and subsequently with Alexa Fluor 488-conjugated anti-rat secondary antibody (Table 1). Pictures were captured on a Ti2 widefield microscope (Nikon, Melville, NY). Images were masked and area was analyzed using ImageJ.

**Table 1:** List of antibodies, fluorophores, clones, usages, and manufacturers.

Antibody	Fluorophore	Clone	Usage	Manufacturer
Rat anti-mouse CD16/CD32	N/A	2.4G2	F_c_ block	BD Biosciences
Mouse anti-mouse CD64	PE	X54-5/7.1	Eye	BioLegend
Hamster anti-mouse CD11c	BV 421	HL3	Eye and Compensation	BD Biosciences
Rat anti-mouse Ly6G	PE-CF594	1A8	Eye	BD Biosciences
Mouse anti-mouse NK1.1	PE-CF594	PK136	Eye	BD Biosciences
Rat anti-mouse Siglec F	PE-CF594	E50-2440	Eye	BD Biosciences
Rat anti-mouse B220	PE-CF594	RA3-6B2	Eye	BD Biosciences
Rat anti-mouse CD8	PE-CF594	53-6.7	Eye a	BD Biosciences
Rat anti-mouse CD4	PE-CF594	RM4-5	Eye and Compensation	BD Biosciences
Rat anti-mouse MHC II	AlexaFluor 700	M5/114.15.2	Eye	BioLegend
Rat anti-mouse CD11b	APC-Cy7	M1/70	Eye and Compensation	BD Biosciences
Rat anti-mouse CD45	PE-Cy7	30-F11	Eye and Compensation	BD Biosciences
Rat anti-mouse CD19	PE	1D3	Compensation	BD Biosciences
Rat anti-mouse CD19	AlexaFluor 700	1D3	Compensation	BD Biosciences
Fixable Viability Dye	eFluor 506	N/A	Eye and Compensation	Invitrogen
Rat anti-mouse CD102 (ICAM2)	N/A	3C4(mIC2/4)	Immunofluorescence	BD Biosciences
Donkey anti-rat (H + L)	AlexaFluor 488	N/A	Immunofluorescence	Invitrogen

### Flow cytometry of whole eyes and ocular tissues

Experiments were performed as previously described^11^. Briefly, mice were sacrificed and eyes were enucleated and stored in cold HBSS. Animals did not undergo systemic perfusion because we previously showed no change in mononuclear phagocyte numbers at steady-state or after laser injury with or without systemic perfusion^11^. Eyes were dissected to remove optic nerve, extraocular muscles, orbital tissue, and conjunctiva. Remaining cornea, sclera, iris, ciliary body, vitreous, retina, and choroid were minced into small pieces. Tissues underwent further mechanical and chemical digestion, and were passed through a fine mesh filter to obtain a single cell suspension. Cell suspensions were stained with live/dead and washed. Cell suspensions were blocked and then stained with fluorescently-conjugated antibodies (Table 1). Both eyes were pooled from one mouse to analyze cells per mouse with counts beads as previously described^11^. For dissected iris, choroid, and retina, tissues were not minced, underwent chemical digestion for 1 h, and then were passed through a fine mesh filter. After the fine mesh filter, dissected and whole eye specimens were treated identically. Samples were run on a modified LSRII (BD Biosciences, San Jose, CA) and analyzed using FlowJo v10.

### Statistical analysis

Flow cytometry comparisons between ocular tissues were made using One-Way ANOVA followed by Sidak’s multiple comparisons test. Comparisons for CNV area were made using Student’s unpaired t-test. Flow cytometry comparisons between wildtype and *Flt3*^*−/−*^ or *Flt3l*^*−/−*^ mice, and over time, were made using the Brown-Forsythe and Welch ANOVA followed by Dunnett’s T3 multiple comparison test due to unequal variances between unlasered and lasered mice.
